# Social Physique Anxiety in College Students: The Role of Study Field, Physical Activity, Physical Self-Perception, and Self-Esteem

**DOI:** 10.3390/sports13040119

**Published:** 2025-04-14

**Authors:** Ioannis Tsartsapakis, Aglaia Zafeiroudi

**Affiliations:** 1 Department of Physical Education and Sport Sciences at Serres, Aristotle University of Thessaloniki, 62100 Serres, Greece; 2 Department of Physical Education and Sport Science, University of Thessaly, 42100 Trikala, Greece

**Keywords:** social anxiety, self-esteem, exercise, self-perception, eating disorders, well-being, university students, body ideals

## Abstract

Young adults, especially students, face challenges that increase social anxiety, including Social Physique Anxiety (SPA). The objective of the present study was to examine SPA among college students across various study programs and explore its associations with exercise, physical self-perception, and self-esteem. A total of 465 students, with an average age of 21.3 years, completed the Social Physique Anxiety Scale, Rosenberg Self-Esteem Scale, and Physical Self-Perception Profile. Demographic information and exercise habits were also collected. The findings indicated that the subject of study was not associated with SPA, regardless of gender. Multiple regression analysis identified physical self-worth, self-esteem, and gender as variables associated with SPA among study groups. Higher levels of physical self-worth and self-esteem were linked to lower SPA levels, and gender had a significant association with SPA. These findings suggest that global and physical self-esteem may play important roles in SPA among college students. Promoting regular physical activity and fostering positive physical self-perception among college students, particularly females, could be beneficial in addressing SPA.

## 1. Introduction

The concept of beauty and the ideal body shape is shaped by various cultural and socio-economic factors, including media, the fitness industry, and marketing [1]. However, this perspective often overlooks the social determinants of health and disregards the physical variations in the human body’s shape, size, and fitness. This assumption that health can be visually perceived perpetuates stereotypes [2]. Contemporary marketing reinforces specific body ideals, promoting a “slim” and “toned” physique for female consumers and a “low-fat”, “muscular”, and “V-shaped” physique for male consumers [3,4].

According to objectification theory, research has shown that exposure to idealized body images in media can contribute to body dissatisfaction and influence dietary patterns and behaviors, including the adoption of hypocaloric diets among young adults [5]. This exposure is also associated with a higher prevalence of eating disorders among this age group, even among individuals without prior health concerns [5,6,7].

The transition to university life brings significant changes, including alterations in social networks, increased academic demands, and exposure to new social norms and body image standards. Young adults, particularly students, often encounter challenges that heighten social anxiety, including Social Physique Anxiety (SPA) [4]. SPA, a construct within self-presentation theory, refers to the anxiety experienced when individuals anticipate negative judgments about their physical appearance [8]. It not only reflects negative self-evaluation but also an individual’s perceived inability to create favorable public impressions based on physical attributes [9].

A substantial body of research has identified a strong association between SPA and lower self-esteem (SE), as well as increased psychological distress [10]. Individuals with elevated SPA levels often exhibit lower SE, as heightened concerns about how their bodies are perceived by others contribute to feelings of inadequacy and diminished self-worth [11]. This dynamic can lead to harmful behaviors, such as disordered eating and excessive exercise [12].

The impact of SE on motivation, functional behavior, life satisfaction, and overall well-being has been widely documented, with these effects remaining consistent throughout life [13]. As Fox [14] indicated, SE and physical self-perception (PSP) are fundamental to mental health and well-being. Sabiston et al. [15], define SE as the emotional and evaluative aspect of the self, whereas self-perception refers to its cognitive dimension. PSP encompasses multiple domains, including perceived sporting ability (SPORT), physical conditioning/fitness and exercise (COND), body attractiveness (BODY), physical strength and muscularity (STREN), and physical self-worth (PSW) [16]. According to Fox [17], PSW represents a broader evaluation of an individual’s physical attributes, extending beyond specific domains, such as athletic ability, body attractiveness, fitness, and physical strength, to provide a more comprehensive assessment of PSP. Research indicates PSW as a critical component of both physical and global SE, shaping how individuals perceive their physical characteristics and their overall sense of self-worth [18,19]. A positive PSP has been shown to correlate with higher SE and lower SPA levels [20].

Moreover, physical activity has been found to reduce SPA while enhancing SE and life satisfaction [21]. Additionally, individuals engaged in competitive sports and exercise programs exhibit lower SPA scores compared to those who do not participate in physical activity [22]. This positive effect is likely attributed to improvements in PSP and mental resilience [23]. Brunet and Sabiston [24] conclude that interventions aimed at reducing SPA may help foster motivation for physical activity and encourage behavior. However, the extent to which exercise impacts SPA levels remains inconclusive.

Given that SPA is rooted in self-presentation concerns and social anxiety, it is understandable that a common approach to managing physique concerns involves behavioral avoidance, meaning avoiding situations likely to provoke anxiety [25]. For instance, a study by Zartaloudi and Christopoulos [26] found that SPA was negatively correlated with physical activity participation and exercise commitment. Similarly, earlier research by Sabiston et al. [27] reported comparable findings. More recently, a study by Tsartsapakis et al. [4] showed that the body mass index (BMI) was positively associated with SPA, whereas SE negatively was associated with SPA in individuals who exercised. Additionally, when individuals are driven by external motivations, such as physical appearance and body improvement to satisfy others, their engagement in exercise tends to decline [26]. Among those who do not participate in physical activity, body dissatisfaction emerged as a significant positive predictor of SPA. According to Alaya et al. [28], individuals concerned about others’ perceptions of their physical capabilities are less inclined to participate in physical activity programs. These findings indicate that exercise both influences and is influenced by SPA, highlighting the complex relationship between body image concerns and physical activity behavior.

Research consistently shows that women exhibit higher levels of SPA than men across various social and age groups [22,29,30,31,32]. Comparative analyses further reveal that men tend to have lower SPA scores than women [29]. Similarly, a study by Portman et al. [31] found that female exercisers reported higher SPA levels compared to their male counterparts. However, the findings of the study suggest that SPA does not necessarily deter body-conscious individuals from engaging in regular physical activity. Hagger and Stevenson [30] also identified a persistent gender discrepancy in SPA levels and physical SE among adolescents. Their study revealed that, except for the 11–12 age group, females consistently exhibited higher SPA and lower physical SE ratings compared to males. These disparities appear to be consistent across different age groups, reinforcing the gendered nature of SPA.

A comprehensive review of the extant literature found no research specifically examining the relationship between SPA, PSP, SE, physical activity, and gender in students whose field of study may or may not be directly related to exercise and their participation or non-participation in exercise programs. The primary objective of the present study is to investigate the predictors of SPA among college students, with a particular emphasis on the mediating effects of physical self-perception, self-esteem, exercise participation, and gender. Furthermore, this study will examine whether the field of study influences SPA levels, considering both exercising and non-exercising populations.

Therefore, the following hypotheses were formulated for this investigation:

(A) Male and female students whose field of study is related to physical activity will have lower SPA than those whose field of study is not related to physical activity.
(B) Exercising females and exercising males will have a lower SPA than their non-exercising counterparts.
(C) SPA will be examined in relation to the independent variables of E, PSW, SPORT, COND, BODY, STREN, BMI, exercise, and gender and across the groups, Physical Education and Sport Studies (PESS), Other Studies Not Related to Exercise (OSNRE), Practitioners (PRs), and Non-Practitioners (NPRs), which will be analyzed separately for males and females. The variable “exercise” will be excluded for the PR and NPR groups.

## 2. Materials and Methods

### 2.1. Study Design and Procedure

A power analysis was conducted using the G*Power software (v. 3.1.9.4, Heinrich-Heine-Universität Düsseldorf, Düsseldorf, Germany) to determine the required sample size for our study. Based on previous research findings [33,34], we estimated the effect size at 0.15. We set the level of significance at α = 0.05. The G*Power analysis yielded the following results: critical *F*-value = 2.0, numerator degrees of freedom = 9, denominator degrees of freedom = 104, and a total required sample size of 114 participants.

In the present study, a correlation design was utilized to explore the factors that are likely to be associated with and predict SPA. In addition, comparisons were made between PESS and OSNRE male and female groups, and the PR and NPR male and female groups to determine their differences in SPA, SE, and PSP so that appropriate results could be extracted. This study was reviewed and granted approval by the Local Ethics Research Committee, School of Physical Education and Sport Science at Serres, Aristotle University of Thessaloniki (ERC-020/2024) prior to data collection. Survey posters were placed at the entrance and exit doors of the universities, and relevant emails were disseminated by the departmental secretariats of the universities. Finally, announcements were disseminated via social media platforms (e.g., Facebook and Instagram) to reach the intended student population. Data collection spanned an eight-week period, from 15 October to 18 December 2024, with the first researcher (I.T. or A.Z.) strategically positioned at the entrance to the university building to ensure the collection of objectives, unbiased data. Prior to participation, all students were informed of the purpose of the survey and provided written consent to the second researcher (I.T. or A.Z.), who was responsible for collecting the questionnaires. In accordance with the protocol of this study, questionnaires that were not fully completed were excluded from the final analysis. Similarly, participants who did not provide written consent to participate were also excluded from the study. A convenience sample was utilized, with any individual aged 18 years and above who was a regular student at a Greek university being eligible to participate in the survey. To be included in the group of trainees, students had to have exercised regularly (minimum three times a week for approximately an hour) for at least two years or more. Conversely, students in the non-exercising group had to have not exercised regularly for at least the last two years.

### 2.2. Participants

The data were collected at a total of 11 universities and 18 schools, and the survey involved 465 participants with an average age of 21.3 years (SD = 2.2 years). The sample included 187 male participants with an average age of 21.2 years (SD = 2.2 years) and 278 female participants with an average age of 21.4 years (SD = 2.2 years). For the purposes of this study, participants were initially divided into two groups: the PESS group, which included students whose academic endeavors were directly associated with physical activity [n = 197, mean age 20.7 years (1.8 years)], and the subsequent group, designated as OSNRE, comprised students whose academic pursuits were not related to physical activity at any level [n = 268, mean age 21.8 years (2.2 years)]. A secondary classification was also made according to the students’ exercise or non-exercise status: PR group [n = 314, mean age 21.2 years (2.1 years)] and NPR group [n = 151, mean age 21.5 years (2.2 years)]. Table 1 presents the data for both the entire sample and the groups that were divided for the purposes of this study.

### 2.3. Measures

#### 2.3.1. Demographics

All participants provided self-reported demographic information, including gender, age, height, weight, university attended, and whether or not they participated in exercise programs. Their ΒΜΙ was calculated by dividing their weight in kilograms by the square of their height in meters. Participants who reported exercising answered three questions regarding the type of exercise they did, the days they exercised per week, and the hours they exercised per day.

#### 2.3.2. Social Physique Anxiety (SPA)

The Social Physique Anxiety Scale (SPAS) [35] was employed to assess SPA. The scale was adapted for the Greek population in terms of its validity and reliability by Psychountaki et al. [36] to enable the measurement of SPA in the students who were examined. The original scale of Hart et al. [35], which comprises 12 items, was utilized. Each item of the SPAS can be responded to on a five-point Likert scale (1 “not at all”, 2 “slightly”, 3 “moderately”, 4 “very”, and 5 “extremely”). Higher scores indicate increased levels of SPA. For an analysis of the scale’s internal consistency in the present study refer to Section 3.2.1.

#### 2.3.3. Self-Esteem (SE)

The Rosenberg Self-Esteem Scale [37] was utilized to evaluate the global SE of the subjects. This questionnaire is regarded as a highly reliable psychometric tool for measuring overall SE, with the scale capturing total SE by directly probing individuals on ten items, with five items presenting positive connotations and five presenting negative connotations. Responses are provided on a four-point Likert scale (from 1 “strongly disagree” to 4 “strongly agree”). Examples of questions include “I feel that I have enough skills” and “I feel worthless sometimes”. According to the scale manufacturer, higher scores indicate higher SE, and the scale is intended for use with individuals over the age of 12. The scale’s validity and reliability were previously assessed in the Greek population by Galanou et al. [38]. For an analysis of the scale’s internal consistency in the present study refer to Section 3.2.1.

#### 2.3.4. Physical Self-Perception (PSP)

The original Physical Self-Perception Profile (PSPP) questionnaire, first developed by Fox and Corbin [39], contains 30 items and consists of five subscales with six items presented in a structured alternative format with a possible score range of 6 to 24. The abridged version of the PSPP, termed the PSPP-15, was developed by Vlachopoulos et al. [16] and was utilized in the present study. The questionnaire under consideration comprises inquiries such as, “In general, I have high levels of endurance and fitness” or “I feel that in general, I can maintain an attractive body”. Scoring is conducted on a seven-point Likert scale, ranging from 1 (“I do not agree at all”) to 7 (“I totally agree”), with higher scores indicating more positive physical self-perceptions. The psychometric properties of the PSPP were introduced by Fox [17] and have since been replicated in numerous published studies. The questionnaire used in the present study was evaluated for its validity and reliability in the Greek population by Vlachopoulos et al. [16]. The internal consistency of the scale was evaluated using Cronbach’s alpha coefficient (refer to Section 3.2.1).

### 2.4. Data Analyses

The data for this study were extracted through the implementation of a statistical analysis, which was carried out using the statistical software IBM SPSS Statistics, version 29.0 (IBM Co., Ltd., Armonk, NY, USA). The normality of the results was ascertained through the use of the Kolmogorov–Smirnov test, which revealed that the data followed a normal distribution. The internal consistency of the SPAS, RSES, and PSPP subscales was determined using Cronbach’s Alpha (see Section 3.2.1). In order to ascertain any discrepancies in SPAS, RSES, and the five subscales of PSPP among the following groups, three independent samples *t*-Tests were conducted, with the groups defined as follows: (a) PESS and OSNRE (male and female) and (b) PR and NPR (male and female). Pearson correlation analysis was utilized to identify associations between SPA, SE, the five subscales of PSPP, BMI, and exercise. Subsequently, a stepwise multiple regression analysis was employed, with SPA designated as the dependent variable and the independent variables comprising SE, the five subscales of PSPP, BMI, exercise, and gender. The threshold of statistical significance was established at *p* < 0.05.

## 3. Results

### 3.1. Descriptives—Somatometric and Demographic Data

As illustrated in Table 1, the somatometric characteristics of the entire sample are displayed, along with the data categorized by gender, practitioner status, and field of study. The data are presented as means and standard deviations for the variables of age (years), weight (kilograms), height (meters), and BMI (kilograms per meter squared). Concurrently, Table 2 presents the means and standard deviations for SPA, the RSES, and the five subscales of the PSPP for all the aforementioned groups, including gender, practitioner status, and field of study.

### 3.2. Parametric Analysis

#### 3.2.1. Reliability Analysis

To assess the internal consistency of the study’s survey instruments, Cronbach’s α coefficient was computed. For the SPAS questionnaire, the resulting Cronbach’s index was α = 0.93. For the five scales of the PSPP questionnaire, the following values were obtained for Cronbach’s coefficient: α = 0.95 for the whole scale, α = 0.84 for the SPORT subscale, α = 0.85 for the COND subscale, α = 0.86 for the BODY subscale, α = 0.89 for the STREN subscale, and α = 0.93 for the PSW subscale. Finally, for the SE scale, Cronbach’s coefficient yielded a value of α = 0.87, thereby indicating satisfactory internal consistency.

#### 3.2.2. Independent Samples *t*-Test

To assess the statistical significance of potential differences in means of the SPAS, PSPP subscales, SE, BMI, and age between men attending PESS and OSNRE, and women attending PESS and OSNRE, as well as between male PRs and NPRs and female PRs and NPRs, four independent samples *t*-Tests were conducted for each pair of groups. The first and second independent samples *t*-Tests were conducted to compare the psychological and physical self-perceptions scores of the PESS-Men and OSNRE-Men groups, as well as the PESS-Women and OSNRE-Women groups (see Table 3 for details). For both men and women, the PESS groups reported significantly higher perceptions in SPORT, COND, and related items (with the exception of SPA, which showed no significant differences). The implications here suggest that members of the PESS groups perceive themselves as more athletically capable and fitter than their OSNRE counterparts.

In the context of body-related perceptions, while BODY scores demonstrated a statistically significant difference among male subjects, this was not observed among female subjects. In the context of BMI and age, it was observed that the PESS group demonstrated lower BMI values and were found to be younger on average in both comparisons. This consistency lends further credence to the hypothesis that these groups may exhibit differences in overall physical status and demographic composition.

The third and fourth independent samples *t*-Tests were conducted to make a comparison between PR-MEN and NPR-MEN, as well as NPR-Women and PR-Women, in various physical, psychological, and physical self-perception scores (see Table 4). For both men and women, the PR groups reported significantly higher perceptions in athletic ability (SPORT), fitness (COND), body attractiveness (BODY), and body power (STREN) compared to NPR groups. This suggests exercisers perceive themselves as more capable and fitter. PR groups also had a higher physical self-worth (PSW) and lower Social Physique Anxiety (SPA) for women. Both PR-Men and PR-Women exhibited lower BMI values, indicating better physical health. Age differences were not significant in either comparison. Regular exercise appears to boost self-perceptions and the overall well-being for both genders.

#### 3.2.3. Pearson Correlation

Given that the *t*-Test analysis did not reveal significant differences between the subgroups of different study fields (PESS—OSNRE) of men and women, a decision was made to perform a correlation analysis between the subgroups of PRs and NPRs of the male and female students separately. This approach was adopted to avoid the error of estimating low or high scores due to gender. Consequently, we conducted four distinct correlation analyses for the subgroups: PR-Men, PR-Women, NPR-Men, and NPR-Women. Table 5, Table 6, Tables 8 and 9 present the Pearson correlation coefficients between various psychological and physical self-perception subscales, and the BMI for all participant subgroups (PR-Men, PR-Women, NPR-Men, and NPR-Women). The correlation strength was classified according to Wassertheil and Cohen [40]: weak (r = 0.10–0.29), moderate (r = 0.30–0.49), and strong (r ≥ 0.50).

Statistically significant correlations are highlighted, indicating the strength and direction of relationships between these variables. Notably, SPA shows moderate to strong negative correlations with several key physical self-perception subscales.

The description of the results of the correlation analysis in Table 5 and Table 6 is briefly shown in Table 7. This exposition will be followed by an overview of the results obtained from the PR-Men and PR-Women groups.

The key similarities are as follows: Female trainees demonstrate slightly stronger positive relationships between self-esteem and perceptions of physical attributes. SE positively correlates with perceptions of PSW and BODY, regardless of gender.

The key differences are as follows: Female trainees demonstrate slightly stronger positive relationships between SE and perceptions of physical attributes. BMI plays a more pronounced positive role in SPA for male trainees.

The description of the results of the correlation analysis in Table 8 and Table 9 is briefly shown in Table 10. This exposition will be followed by an overview of the results obtained from the NPR-Men and NPR-Women groups.

The key similarities are as follows: Both groups exhibit strong negative correlations between SPA and perceptions of PSW and BODY. SE is positively correlated with perceptions of PSW and BODY, irrespective of gender.

The key differences are as follows: Female sedentary students show a positive correlation between BMI and SPA, whereas male sedentary students exhibit no significant BMI impact. Female sedentary students display slightly weaker positive links between SE and perceptions of physical attributes compared to male sedentary students.

A comprehensive comparison of the correlation analyses performed on PR and NPR is presented in Table 11.

The key differences between and insights on the two groups is summarized as follows:

1. Physical Activity Benefits: PRs of both genders show more positive SE associations and a less pronounced negative role of BMI compared to their NPR counterparts. Physical activity seems to produce some positive impacts on perceptions of PSW and BODY.
2. Gender Variations: Female trainees and sedentary females tend to display slightly stronger negative correlations between SPA and PSW, suggesting a heightened sensitivity to the PSP.
3. BMI’s Role: BMI plays a more positive role in male trainees’ SPA but tends to negatively influence the PSP in sedentary females.

#### 3.2.4. Multiple Regression Analysis (Stepwise)

A multiple regression analysis was performed to test the third hypothesis, which concerns the prediction of SPA by the independent factors of SE, PSW, SPORT, COND, BODY, STREN, BMI, exercise, and gender, respectively, for each group of (a) PESS students, (b) OSNRE students, (c) PR participants, and (d) NPR participants. This analysis was performed stepwise (see Table 12). For the PESS group, the overall model was statistically significant (R^2^ = 0.690, F_(5.196)_ = 85.181, *p* < 0.001). The tolerance and VIF values were as follows: PSW (0.203, 4.918), SE (0.621, 1.611), gender (0.882, 1.133), BODY (0.245, 4.083), and STREN (0.553, 1.809). Variables such as PSW, SE, and gender were associated with SPA (see Table 10). Consequently, the hypothesis for the PESS group was partially supported, with the model explaining 69.0% of the variance in SPA (R^2^ = 0.690).

For the OSNRE group, the overall model was statistically significant (R^2^ = 0.635, F_(4.266)_ = 114.002, *p* < 0.001). The tolerance and VIF values were as follows: PSW (0.201, 4.985), gender (0.913, 1.095), SE (0.510, 1.961), and BODY (0.206, 4.853). Variables such as PSW, SE, and gender were identified as being associated with SPA (see Table 10). The hypothesis for the OSNRE group was supported, with the model explaining 63.5% of the variance in SPA (R^2^ = 0.635).

For the PR group, the overall model was statistically significant (R^2^ = 0.678, F_(5.313)_ = 129.508, *p* < 0.001). The tolerance and VIF values were as follows: PSW (0.243, 4.107), gender (0.938, 1.066), BODY (0.269, 3.713), SE (0.599, 1.671), and STREN (0.581, 1.722). Multiple variables, including PSW, SE, BODY, and gender, were associated with SPA (see Table 10). Consequently, the hypothesis for the PR group was partially corroborated, with the model explaining 67.8% of the variance in SPA (R^2^ = 0.678).

For the NPR group, the overall model was statistically significant (R^2^ = 0.626, F_(3.149)_ = 81.523, *p* < 0.001). The tolerance and VIF values were as follows: PSW (0.546, 1.830), SE (0.515, 1.944), and gender (0.921, 1.086). Yet again, SPA was associated with variables such as PSW, SE, and gender (see Table 10). Consequently, the hypothesis for the NPR group was supported, with the model explaining 62.6% of the variance in SPA (R^2^ = 0.626).

## 4. Discussion

The primary aim of this study was to examine the factors associated with SPA among college students, focusing on the roles of PSP, SE, and exercise participation. Specifically, this study aimed to explore how these factors are linked to SPA in students enrolled in PESS programs compared to those in non-related fields, while also investigating differences between students who engage in regular exercise and those who do not. This study was guided by three hypotheses: (1) male and female students whose field of study is related to physical activity will have lower SPA than those whose field of study is not related to physical activity, (2) exercising males and females will have lower SPA than their non-exercising counterparts, and (3) SPA will be associated with SE, SPORT, COND, BODY, STREN, PSW, BMI, exercise, and gender within each group.

### 4.1. Relations of the Study Field with SPA, PSP, and SE

Contrary to our initial hypothesis, the results indicated no significant differences in SPA scores between PESS and OSNRE students for both genders. This finding suggests that academic involvement in physical activity is not necessarily associated with reduced concerns about one’s physique in social settings. Our findings align with those of a previous study by Tsartsapakis et al. [4], which showed that students whose academic studies were directly related to physical education and exercise did not exhibit differences in SPA compared to students from other departments whose studies were unrelated to physical education and exercise.

The lack of significant differences in SPA between PESS and OSNRE students challenges the assumption that education and training in physical activity environments inherently alleviates physique-related anxieties. Students enrolled in physical education courses may encounter augmented pressures related to their awareness of physical health and fitness. This heightened awareness could result in greater self-scrutiny and expectations to embody the physical ideals emphasized in their academic pursuits, potentially sustaining or amplifying physique-related stress (SPA). Research indicates that individuals in environments that place significant emphasis on physical appearance and performance may experience elevated SPA due to these pressures [41,42,43]. Consequently, the anticipated protective effect of studying physical activity may be mitigated by these additional factors.

Young adults are frequently exposed to idealized body images through television, magazines, and, most notably, social media platforms [44,45]. These media often promote unrealistic and narrow standards of beauty and fitness, which can exacerbate body dissatisfaction and SPA [46]. Holland and Tiggemann [47] found that the use of social networking sites is associated with increased social comparison, contributing to body image concerns and physique-related anxieties. The interactive nature of these platforms enables immediate feedback and comparison, intensifying the impact on body image concerns. Both PESS and OSNRE students are equally susceptible to these influences, as media consumption patterns are generally similar among university students regardless of their academic focus.

Given the powerful effects of age-related developmental challenges and media influence, it is plausible that these factors overshadow any potential differences in SPA that might arise from academic specialization. While the present study did not examine the effect of social media as a factor influencing SPA in young adults, this could be a subject for future research. Despite PESS students’ engagement in physical activity, societal pressures to meet certain physical ideals during this life stage may lead to the homogenization of SPA levels across disciplines. While the sample population did not exhibit a significant difference in SPA, the study results indicated that PESS students demonstrated higher scores than OSNRE students on several subscales of the PSPP, including SPORT, COND, STREN, BODY, and PSW (significant for males). These findings imply that PESS students have more positive perceptions of their physical abilities and overall physical self-perception. This aligns with the theoretical framework proposed by Fox [48], who suggested that exercise interventions can lead to improvements in PSP and SE.

For women, significant differences in SPORT, COND, and STREN suggest that PESS women perceive themselves as more athletically and physically competent than OSNRE women. This aligns with findings by Dishman et al. [49] and Opdenacker et al. [50], who demonstrated that increased physical activity and participation in sports are associated with higher PSP and SE. However, the findings in PSW for women (*p* = 0.052) were close to being statistically significant, possibly indicative of societal norms and cultural factors that place a greater emphasis on appearance for women. These norms may influence how women perceive and report body-related perceptions, resulting in a heightened pressure to conform to specific beauty standards [51].

Finally, with regard to SE, the stable SE scores across the PESS-Men and OSNRE-Men study fields suggest that factors other than physical education play a more substantial role in shaping male SE during university years [19,50]. Interventions aimed at enhancing male SE may need to address broader aspects of the self-concept and personal achievements. For women, the significant positive association between physical education and female SE underscores the value of promoting physical activity among women. Educational programs that foster female participation in sports and physical activities can contribute to a higher SE and overall well-being [52,53].

The second hypothesis posited that females and males engaging in physical activity would demonstrate lower SPA scores compared to their non-exercising counterparts. The results of this study offer a nuanced understanding of this hypothesis, revealing significant differences for females but not for males. For females, the findings indicated a substantial difference in SPA scores between PR-Women and NPR-Women, with PR-Women exhibiting lower SPA scores. This suggests that physical activity is associated with reduced SPA among female college students. These results partially align with the research conducted by Mülazımoğlu-Ballı et al. [22], which identified associations between regular exercise and lower levels of SPA compared to individuals who do not engage in physical activity.

Conversely, among male students, there was no significant difference in SPA scores between the PR-MEN and NPR-MEN groups, suggesting that physical activity is not strongly associated with SPA levels in male students. The observed reduction in SPA among exercising females is consistent with existing literature suggesting that physical activity may enhance body image and reduce physique-related anxiety in women. The potential mechanisms underlying these associations could include improvements in bodily autonomy and self-efficacy, which contribute to enhanced self-perceptions and reduced concerns about social evaluations [54,55]. These phenomena could be further influenced by societal pressures regarding body image that women often encounter [56,57]. Such pressures may intensify physique-related concerns, highlighting the importance of understanding the complex interplay between physical activity, societal influences, and SPA in women.

### 4.2. Relations of Physical Activity (Practitioners and Non-Practitioners) with SPA, PSP, and SE and Gender Differences

The absence of substantial differences in SPA among males may be attributed to societal expectations and pressures pertaining to body image. Males often encounter reduced societal scrutiny concerning their physiques, which may contribute to comparatively lower SPA levels regardless of their physical activity status [58,59]. Furthermore, baseline SPA scores for men were found to be lower compared to those observed in women, suggesting that SPA might be less prominent among male students [30].

Although this study did not find significant differences in SPA among males based on activity level, it did reveal significant variations across several domains of PSP, including SPORT, COND, BODY, STREN, and PSW. A similar pattern was observed among females, with PR-Women demonstrating higher scores across these domains: SPORT, COND, BODY, STREN, PSW, and SE. Among PR-Women, the observed lower levels of SPA and higher levels of SE appear to be associated with these enhanced PSPs. This suggests a potential relationship where physical activity is linked to improved self-perceptions, which may support higher SE and lower SPA [60]. The differential findings between genders suggest that the interplay between physical activity, SPA, and SE may function differently for men and women. For women, physical activity seems to be associated with enhanced self-perceptions, which correlate with elevated SE and lower SPA. Conversely, for men, while PSP improves with physical activity, this does not appear to be strongly associated with SE or SPA, pointing to potential moderating variables or alternative pathways. Societal norms often encourage men to derive self-worth from achievements in broader life domains, such as career success, financial stability, or social status, which may reduce the influence of PSP on overall SE [61].

### 4.3. Factors Associated with SPA

The third hypothesis posited that SPA would be associated with a combination of PSP variables (PSW, SPORT, COND, BODY, and STREN), as well as SE, BMI, exercise, and gender. These associations were examined across four distinct groups: PESS, OSNRE, PR, and NPR. For the PR and NPR groups, the variable “exercise” was excluded from the analysis.

The findings of this study partially supported this hypothesis, revealing that certain psychological and demographic factors are significantly associated with SPA among college students, with some variation across different groups.

Among the PR group, SPA was associated with perceived PSW, gender, BODY, SE, and STREN. The inclusion of STREN in this group may reflect the importance placed on physical capabilities among individuals who engage regularly in physical activity. Gender differences were also significant, suggesting that the experience of SPA varies based on gender among physically active students.

In the NPR group, SPA was significantly associated with perceived PSW, SE, and gender. Notably, perceived PSW had a particularly strong association with SPA in this group, indicating that for students who are less physically active, their overall sense of physical worth plays an important role in their levels of SPA.

In the PESS group, SPA was associated with higher levels of PSW, SE, gender, BODY, and STREN. The findings of this study indicate that students who possess a high degree of confidence in their physical abilities and overall self-worth demonstrate lower levels of SPA in social settings. Gender differences also emerged as significant, suggesting variation in SPA experiences between male and female students in this group. Additionally, this study found that STREN was a contributing factor, indicating that students’ perceptions of their physical strength are linked to their levels of SPA.

A similar pattern was observed for students in the OSNRE group, with the exception of the STREN factor. Higher perceived PSW, SE, and BODY were associated with lower SPA. The absence of the STREN factor is consistent with the low rate of exercise among students in the OSNRE group, indicating that this factor may not contribute positively to SPA reduction in this context. Gender differences were significant again, highlighting variations in SPA experiences based on gender across diverse fields of study.

These findings align with previous research emphasizing the impact of PSP and SE on SPA [4,32,62,63]. Individuals with higher SE and positive perceptions of their physical selves are generally less susceptible to anxiety about how others view their bodies. This relationship underscores the protective role that a positive self-image may play in mitigating SPA.

The consistent influence of gender across all groups suggests that SPA experiences differ between male and female students [29,30,31]. This outcome aligns with studies indicating that societal and cultural factors can contribute to variations in body image concerns [64,65]. Understanding these differences is essential for developing targeted interventions that address the specific needs of diverse student populations.

While variables such as SPORT, COND, BMI, and exercise showed relationships with SPA, they did not consistently emerge as significant across all groups. This inconsistency suggests that while these factors are related to one’s PSP, their direct association with SPA may be influenced by other psychological factors.

The findings of the present study demonstrate that SPA among college students is influenced by a dynamic interplay of psychological and physical factors, thereby supporting the final hypothesis. The replicability of these associations across diverse groups highlights the collective role of psychological constructs, such as SE and PSP, and physical attributes, including gender, in relation to SPA. These findings emphasize the need for comprehensive interventions that address both psychological well-being and physical health to mitigate SPA effectively. Strategies that enhance SE and PSP, such as counseling services and body image workshops, could be beneficial—especially for females. Additionally, encouraging regular physical activity is crucial, as it not only improves physical health but also fosters a positive body image.

### 4.4. Study Limitations

While our study provides valuable insights, several limitations must be acknowledged. First, the use of convenience sampling might have introduced a selection bias, as participants who are more interested in physical activity or have specific attitudes toward body image may have been more likely to participate. This could limit the representativeness of our sample. Second, this study relied on self-reported data, which are susceptible to social desirability bias and inaccuracies in recall. Participants may have overestimated or underestimated their physical activity levels, SE, or PSP.

Third, this study did not account for other potential confounding variables, such as socio-economic status, mental health status, or previous experiences with body image issues. These factors could have influenced SPA and its associations with other variables, and their exclusion might have led to an incomplete understanding of the relationships examined. Fourth, this study did not differentiate between types and intensities of physical activities. Different forms of exercise (e.g., team sports, individual workouts, and yoga) may have varying effects on SPA and related variables, and future research should explore these nuances.

Additionally, our study sample was limited to university students in Greece, which may restrict the generalizability of our results to other populations. Replicating this study in different cultural and educational contexts would enhance the robustness and applicability of the findings. Furthermore, the cross-sectional nature of this study imposes constraints on the interpretation of our findings. As the reviewer highlighted, this design limits our ability to infer directionality or causation. For instance, while we found that higher SE and positive PSP are associated with lower SPA, it is also plausible that lower SPA contributes to higher SE and better PSP. Although the statistical terminology used in regression analyses may include “predictors”, we acknowledge that these relationships should be interpreted as associative rather than causal or predictive in nature.

To provide greater clarity on these issues, longitudinal studies are required to examine the temporal dynamics of these variables and establish directionality more robustly. Addressing these limitations in future research will help build a more comprehensive and generalizable body of evidence regarding the factors influencing SPA and the effectiveness of interventions aimed at reducing it.

### 4.5. Implications of This Study

Our findings underscore the importance of physical activity, positive PSP, and SE in reducing SPA among college students. Universities and educational institutions should promote regular physical activity and develop targeted interventions to address gender-specific body image concerns. Creating a supportive and inclusive campus environment that celebrates body diversity and fosters self-confidence can significantly mitigate SPA and enhance the overall mental well-being of students. Also, our findings highlight the multifaceted nature of SPA, emphasizing both physical and psychological contributors. The consistent significance of body attractiveness perceptions and gender suggests that interventions aiming to reduce SPA should prioritize improving body image and addressing weight-related concerns, especially among females. The enhancement of SE and PSW, particularly among non-exercising students and females, has the potential to serve as an effective strategy for mitigating SPA.

## 5. Conclusions

This study provides valuable insights into SPA among college students, revealing that academic involvement in physical activity is not necessarily associated with reduced physique-related concerns. The findings indicate no significant differences in SPA scores between PESS and OSNRE students, challenging the assumption that education in physical activity environments is inherently linked to lower physique-related anxieties. Additionally, female students who engage in regular exercise exhibit lower SPA scores compared to their non-exercising counterparts, whereas no such difference is observed among male students.

This study highlights the association of physical activity with lower SPA and enhanced PSP among female students, emphasizing the need for targeted interventions that encourage physical activity among women. The consistent associations between PSP variables and SE with SPA underscore the importance of psychological well-being in addressing physique-related anxieties.

Importantly, this study contributes to the existing literature by demonstrating that the role of physical education curricula in relation to SPA is not universal. It sheds light on the complex interplay between physical activity, self-perception, gender, and societal influences, such as social media, and emphasizes the need for a deeper understanding of these relationships.

In conclusion, the dynamic interaction of psychological and physical factors plays an important role in shaping SPA among college students. Holistic interventions that address both psychological well-being and physical health are crucial for reducing SPA, with a particular focus on enhancing SE and PSP and fostering regular physical activity.

## Figures and Tables

**Table 1:** Somatometric characteristics of the entire sample and the data categorized by gender, practitioner status, and field of study.

		Age (Years)	Hight (cm)	Weight (kg)	BMI (kg/m^2^)
	N	M ± SD	M ± SD	M ± SD	M ± SD
Total Sample	465	21.3 ± 2.2	172 ± 9.0	68.4 ± 13	23.0 ± 3.3
Total Men	187	21.2 ± 2.2	180 ± 7.0	78.5 ± 11	24.2 ± 3.0
Total Women	278	21.4 ± 2.2	167 ± 6.0	61.6 ± 10	22.1 ± 3.2
PR Men	142	21.1 ± 2.2	180 ± 7.0	77.9 ± 11	23.9 ± 3.0
PR Women	172	21.4 ± 2.1	167 ± 7.0	61.0 ± 9.0	21.8 ± 2.6
NPR Men	45	21.7 ± 2.0	179 ± 6.0	80.3 ± 11	25.0 ± 3.3
NPR Women	106	21.5 ± 2.4	166 ± 7.0	62.6 ± 11	22.8 ± 3.9
PESS Men	92	20.4 ± 1.6	181 ± 8.0	78.1 ± 12	23.8 ± 2.8
PESS Women	105	21.0 ± 2.0	166 ± 6.0	59.5 ± 8.0	21.5 ± 2.4
OSNRE Men	95	22.0 ± 2.4	179 ± 6.0	78.8 ± 11	24.6 ± 3.5
OSNRE Women	173	21.7 ± 2.3	167 ± 6.0	62.9 ± 11	22.5 ± 3.5

M = mean, SD = standard deviation, N = number of participants, PR = Practitioners, NPR = Non-Practitioners, PESS = Physical Education and Sport Studies, and OSNRE = Other Studies Not Related with Exercise.

**Table 2:** Descriptives for SPA, SE, and five subscales of PSPP including gender, practitioner status, and field of study.

Group		SPA	SPORT	BODY	COND	STREN	PSW	SE
	N	M ± SD	M ± SD	M ± SD	M ± SD	M ± SD	M ± SD	M ± SD
Total M	187	24.0 ± 9.0	5.16 ± 1.2	4.84 ± 1.2	5.00 ± 1.4	4.82 ± 1.2	5.01 ± 1.3	30.7 ± 5.3
Total W	278	30.7 ± 10.4	4.69 ± 1.5	4.68 ± 1.3	4.48 ± 1.4	4.64 ± 1.4	4.66 ± 1.4	29.2 ± 5.3
PR M	142	23.6 ± 8.3	5.40 ± 1.2	5.00 ± 1.7	5.38 ± 1.6	5.00 ± 1.2	5.27 ± 1.2	30.7 ± 5.1
PR W	172	29.2 ± 10.0	5.21 ± 1.2	4.97 ± 1.2	5.14 ± 1.1	5.14 ± 1.2	4.98 ± 1.2	30.1 ± 5.3
NPR M	45	25.4 ± 10.7	4.47 ± 1.0	4.41 ± 1.3	3.80 ± 1.2	4.33 ± 1.4	4.56 ± 1.3	30.9 ± 5.8
NPR W	106	33.0 ± 10.8	3.84 ± 1.5	4.20 ± 1.4	3.40 ± 1.2	3.83 ± 1.4	4.15 ± 1.5	27.8 ± 4.9
PESS M	92	23.0 ± 7.6	5.51 ± 1.0	5.15 ± 1.1	5.39 ± 1.1	5.04 ± 1.3	5.43 ± 1.0	30.7 ± 5.2
PESS W	105	29.4 ± 11.0	5.33 ± 1.1	4.85 ± 1.3	5.19 ± 1.1	5.09 ± 1.2	4.87 ± 1.3	30.2 ± 5.2
OSM	95	25.0 ± 10.0	4.81 ± 1.3	4.54 ± 1.3	4.61 ± 1.4	4.60 ± 1.4	4.76 ± 1.4	30.8 ± 5.3
OSW	173	31.5 ± 10.1	4.30 ± 1.5	4.57 ± 1.3	4.04 ± 1.5	4.37 ± 1.4	4.53 ± 1.4	28.5 ± 5.2

N = number of participants, SPA = Social Physique Anxiety, SPORT = PSP athletic ability, BODY = PSP body attractiveness, COND = PSP fitness perception, STREN = PSP body power, PSW = PSP physical self-worth, SE = self-esteem, PRs = Practitioners, NPRs = Non-Practitioners, PESS = Physical Education and Sport Studies, OS = OSNRE (Other Studies Not Related to Exercise), M = Men, and W = Women.

**Table 3:** *t*-Test between PESS-Men and OSNRE-Men groups, as well as PESS-Women and OSNRE-Women groups.

	PESS-Men/OSNRE-Men				PESS-Women/OSNRE-Women			
Variable	*t* (df)	*p*-Value	Mean Dif.	95% CI	*t* (df)	*p*-Value	Mean Dif.	95% CI
SPA	*t*(175.21) = −1.528	0.128	−1.99	[−4.56, 0.58]	*t*(276) = −1.603	0.110	−2.07	[−4.62, 0.47]
SPORT	*t*(176.86) = 4.069	<0.001	0.70	[0.36, 1.04]	*t*(269.43) = 6.664	<0.001	1.04	[0.73, 1.35]
COND	t(185) = 4.071	<0.001	0.77	[0.40, 1.14]	t(267.48) = 7.594	<0.001	1.16	[0.86, 1.45]
BODY	*t*(181.97) = 3.525	<0.001	0.61	[0.27, 0.95]	*t*(276) = 1.672	0.096	0.27	[−0.05, 0.60]
STREN	*t*(185) = 2.272	0.012	0.44	[0.06, 0.81]	*t*(242.51) = 4.464	<0.001	0.72	[0.40, 1.04]
PSW	*t*(172.64) = 3.664	<0.001	0.66	[0.31, 1.02]	*t*(276) = 1.953	0.052	0.33	[−0.003, 0.66]
SE	*t*(185) = −0.066	0.947	−0.05	[−1.57, 1.47]	*t*(275) = 2.599	0.010	1.68	[0.41, 2.95]
BMI	*t*(182.69) = −1.945	0.027	−0.85	[−1.72, −0.01]	*t*(270.63) = −2.964	0.002	−1.05	[−1.75, −0.35]
Age	*t*(160.84) = −5.253	<0.001	−1.56	[−2.14, −0.97]	*t*(248.74) = −3.163	0.002	−0.82	[−1.33, −0.31]

Dif. = Difference, CI = Confidence Interval, PESS = Physical Education and Sport Studies, and OSNRE = Other Studies Not Related to Exercise.

**Table 4:** *t*-Test between PR-Men and NPR-Men groups, as well as PR-Women and NPR-Women groups.

	PR-Men/NPR-Men				PR-Women/NPR-Women			
Variable	*t* (df)	*p*-Value	Mean Dif.	95% CI	*t* (df)	*p*-Value	Mean Dif.	95% CI
SPA	*t*(185) = −1.137	0.257	−1.74	[−4.77, 1.28]	*t*(276) = −2.99	0.002	−3.81	[−6.32, −1.30]
SPORT	*t*(185) = 4.557	<0.001	0.91	[0.52, 1.31]	*t*(180.71) = 8.03	<0.001	1.37	[1.03, 1.71]
COND	*t*(185) = 7.887	<0.001	1.58	[1.18, 1.97]	*t*(276) = 12.07	<0.001	1.74	[1.45, 2.02]
BODY	*t*(185) = 2.754	0.003	0.56	[0.16, 0.97]	*t*(276) = 4.92	<0.001	0.77	[0.47, 1.08]
STREN	*t*(185) = 2.890	0.002	0.64	[0.20, 1.08]	*t*(197.82) = 8.29	<0.001	1.31	[1.00, 1.62]
PSW	*t*(185) = 3.319	<0.001	0.71	[0.29, 1.13]	*t*(191.88) = 4.91	<0.001	0.83	[0.50, 1.16]
SE	*t*(185) = −0.277	0.782	−0.25	[−2.03, 1.53]	*t*(275) = 3.60	<0.001	2.30	[1.04, 3.55]
BMI	*t*(185) = −2.000	0.047	−1.03	[−2.04, −0.01]	*t*(161.75) = −2.34	0.010	−1.00	[−1.83, −0.16]
Age	*t*(185) = −1.650	0.101	−0.61	[−1.34, 0.12]	*t*(276) = −0.33	0.372	−0.09	[−0.63, 0.45]

Dif. = Difference, CI = Confidence Interval, PRs = Practitioners, and NPRs = Non-Practitioners.

**Table 5:** Pearson correlation coefficients for PR-Men Group.

N = 142	SPA	SPORT	COND	BODY	STREN	PSW	SE	BMI
SPA	1							
SPORT	−0.403 **	1						
COND	−0.521 **	0.720 **	1					
BODY	−0.692 **	0.609 **	0.681 **	1				
STREN	−0.325 **	0.637 **	0.593 **	0.646 **	1			
PSW	−0.675 **	0.663 **	0.669 **	0.848 **	0.591 **	1		
SE	−0.539 **	0.394 **	0.389 **	0.464 **	0.415 **	0.520 **	1	
BMI	0.390 **	−0.092 **	−0.220 **	−0.299 **	0.181 *	−0.286 **	−0.072	1

Note: ** Correlation is significant at 0.01 level (2-tailed). * Correlation is significant at 0.05 level (2-tailed). N = number of participants and PRs = Practitioners.

**Table 6:** Pearson correlation coefficients for PR-Women Group.

N = 172	SPA	SPORT	COND	BODY	STREN	PSW	SE	BMI
SPA	1							
SPORT	−0.326 **	1						
COND	−0.249 **	0.778 **	1					
BODY	−0.762 **	0.441 **	0.445 **	1				
STREN	−0.408 **	0.700 **	0.763 **	0.591 **	1			
PSW	−0.770 **	0.453 **	0.444 **	0.842 **	0.626 **	1		
SE	−0.678 **	0.310 **	0.359 **	0.629 **	0.512 **	0.703 **	1	
BMI	0.276 **	−0.256 **	−0.167 *	−0.349 **	−0.025	−0.328 **	−0.120	1

Note: ** Correlation is significant at 0.01 level (2-tailed). * Correlation is significant at 0.05 level (2-tailed). N = number of participants and PRs = Practitioners.

**Table 7:** Comparison of correlation analyses between trainees’ groups (PR-Men vs. PR-Women).

	PR-Men	PR-Women
**SPA:**	Strong negative correlations with PSW (−0.675 **) and BODY (−0.692 **). Positive correlation with BMI (r = 0.390 **).	Similar strong negative correlations with PSW (−0.770 **) and BODY (−0.762 **). Positive BMI correlation (r = 0.276 **).
**SE:**	Moderate positive links with PSW (r = 0.520 **) and BODY (r = 0.464 **).	Strong positive links with PSW (r = 0.703 **) and BODY (r = 0.629 **).
**BMI:**	Positive correlation with SPA (r = 0.390 **) but weaker correlations with BODY and PSW.	Similar trends, with BMI negatively linked to PSW (−0.328 **) and BODY (−0.349 **).
**COND and SPORT:**	Moderate negative correlations with SPA. Positive links with SE.	Similar patterns, but slightly stronger positive correlations with SE.

SPA = Social Physique Anxiety, SE = self-esteem, BMI = Body Mass Index, PRs = Practitioners, COND = perceived physical conditioning/fitness and exercise, SPORT = perceived athletic ability, and PSW = perceived physical self-worth. ** Correlation is significant at 0.01 level (2-tailed).

**Table 8:** Pearson correlation coefficients for NPR-Men group.

N = 172	SPA	SPORT	COND	BODY	STREN	PSW	SE	BMI
SPA	1							
SPORT	−0.449 **	1						
COND	−0.460 **	0.794 **	1					
BODY	−0.709 **	0.507 **	0.549 **	1				
STREN	−0.684 **	0.561 **	0.663 **	0.794 **	1			
PSW	−0.670 **	0.545 **	0.655 **	0.934 **	0.771 **	1		
SE	−0.685 **	0.578 **	0.504 **	0.838 **	0.654 **	0.803 **	1	
BMI	0.135	−0.020	−0.134	−0.234	−0.002	−0.344 *	−0.263	1

Note: ** Correlation is significant at 0.01 level (2-tailed). * Correlation is significant at 0.05 level (2-tailed). N = number of participants and NPRs = Non-Practitioners.

**Table 9:** Pearson correlation coefficients for NPR-Women Group.

N = 172	SPA	SPORT	COND	BODY	STREN	PSW	SE	BMI
SPA	1							
SPORT	−0.388 **	1						
COND	−0.418 **	0.792 **	1					
BODY	−0.733 **	0.425 **	0.539 **	1				
STREN	−0.507 **	0.550 **	0.613 **	0.483 **	1			
PSW	−0.772 **	0.445 **	0.544 **	0.909 **	0.590 **	1		
SE	−0.621 **	0.412 **	0.388 **	0.632 **	0.527 **	0.613 **	1	
BMI	0.286 **	−0.348 **	−0.484 **	−0.401 **	−0.022	−0.336 **	−0.180	1

Note: ** Correlation is significant at the 0.01 level (2-tailed). N = Number of participants, NPR = Non-Practitioners.

**Table 10:** Comparison of correlation analyses between Non-Practitioners groups (NPR-Men vs. NPR-Women).

	NPR-Men	NPR-Women
**SPA:**	Strong negative correlations with PSW (−0.670 **) and BODY (−0.709 **). BMI not significantly correlated.	Strong negative correlations with *PSW* (−0.772 **) and BODY (−0.733 **). Positive BMI correlation (r = 0.286 **).
**SE:**	Strong positive links with PSW (r = 0.803 **) and BODY (r = 0.838 **). No BMI impact.	Similar strong positive links with PSW (r = 0.613 **) and BODY (r = 0.632 **). No significant BMI correlation.
**BMI:**	Mostly non-significant correlations with other variables.	Negative correlations with PSW (−0.336 **) and BODY (−0.401 **).
**COND and SPORT:**	Moderate negative correlations with SPA. Positive relationships with SE.	Similar trends, but slightly stronger negative correlations with SPA.

SPA = Social Physique Anxiety, SE = self-esteem, BMI = Body Mass Index, PRs = Practitioners, COND = perceived physical conditioning/fitness and exercise, SPORT = perceived athletic ability, and PSW = perceived physical self-worth. ** Correlation is significant at 0.01 level (2-tailed).

**Table 11:** Comparison of correlation analyses between Practitioners and Pon-Practitioners groups overall.

	PR (Men and Woman)	NPR (Men and Women)
**SPA:**	Strong negative correlations with perceptions of PSW and BODY. BMI plays a positive role in Social Physique Anxiety.	Similar negative correlations, but BMI has a weaker role in males and more pronounced negative correlations in females.
**SE:**	SE positively linked to perceptions of PSW and BODY, with slightly stronger correlations in females.	SE is similarly linked to perceptions of physical self-perceptions but tends to be stronger overall for males.
**BMI:**	BMI positively correlates with SPA (both genders).	BMI plays a weaker or mixed role in males but shows stronger negative effects in females.

SPA = Social Physique Anxiety, SE = self-esteem, BMI = Body Mass Index, PRs = Practitioners, COND = perceived physical conditioning/fitness and exercise, SPORT = perceived athletic ability, and PSW = perceived physical self-worth.

**Table 12:** Multiple regression analysis (stepwise) for associations of SPA for all groups (PESS, OSNRE, PR, and NPR) across both genders.

Variable	PESS		OSNRE		PR		NPR	
	Beta	*p*-Value	Beta	*p*-Value	Beta	*p*-Value	Beta	*p*-Value
PSW	−0.296	0.001	−0.339	0.001	−0.294	0.001	−0.542	0.001
SE	−0.285	0.001	0.103	0.001	−0.273	0.001	−0.260	0.001
Gender	0.175	0.001	0.854	0.001	0.222	0.001	0.169	0.001
BODY	−0.437	0.001	0.654	0.001	−0.438	0.001		
STREN	−0.225	0.001			−0.221	0.001		
**R**	0.831		0.797		0.823		0.791	
**Adjusted R**	0.682		0.630		0.672		0.619	
**R^2^**	0.690		0.635		0.678		0.626	
**F** (**df**)	5.196		4.266		5.313		3.149	
***p*-value**	0.001		0.001		0.001		0.001	

Dependent variable: SPA. PSW = physical self-worth, SE = self-esteem, BODY = body attractiveness, STREN = physical strength and muscularity, PESS = Physical Education and Sport Studies, OSNRE = Other Study Not Related to Exercise, PRs = Practitioners, and NPRs = Non-Practitioners.

## Data Availability

The data presented in this study are available upon request from the corresponding author, due to ethical and privacy reasons.

## Abbreviations

The following abbreviations are used in this manuscript:

SPA	Social Physique Anxiety
SE	Self-esteem
PSP	Physical self-perception
SPORT	Perceived sporting ability
COND	Perceived physical conditioning/fitness and exercise
BODY	Perceived body attractiveness
STREN	Perceived physical strength and muscularity
PSW	Perceived self-worth
BMI	Body Mass Index
PESS	Physical Education and Sports Studies
OSNRE	Other Studies Not Related to Exercise
PRs	Practitioners
NPRs	Non-Practitioners
